# Comparison of the diagnostic efficacy of systemic inflammatory indicators in the early diagnosis of ovarian cancer

**DOI:** 10.3389/fonc.2024.1381268

**Published:** 2024-07-02

**Authors:** Liyun Song, Qi Wu, Suning Bai, Jing Zhao, Jie Qi, Junmei Zhang

**Affiliations:** Department of Gynecology, Hebei General Hospital, Shijiazhuang, China

**Keywords:** ovarian cancer, systemic immune-inflammation index, prognostic nutritional index, fibrinogen-to-albumin ratio, neutrophil-to-lymphocyte ratio, platelet-to-lymphocyte ratio, monocyte-to-lymphocyte ratio, diagnosis

## Abstract

**Background:**

This study aimed to determine the diagnostic accuracy of CA125, HE4, systemic immune-inflammation index (SII), prognostic nutritional index (PNI), fibrinogen-to-albumin ratio (FAR), neutrophil-to-lymphocyte ratio (NLR), platelet-to-lymphocyte ratio (PLR), monocyte-to-lymphocyte ratio (MLR), and the combination of the six inflammatory-nutritional markers for ovarian cancer (OC) to identify the best diagnostic indicator for OC early diagnosis. An extensive study was performed to establish the connection between these indicators and the pathological aspects of OC.

**Methods:**

A total of 170 individuals were included in this study, with 87 diagnosed with OC and 83 with benign ovarian tumors (BOTs). The diagnostic abilities of the variables were evaluated by calculating sensitivity, specificity, and area under the ROC curves. Through the use of DCA, we evaluated the variables’ clinical value in the discrimination of ovarian masses.

**Results:**

All markers showed significant diagnostic power for OC. CA125, HE4, SII, FAR, and MLR levels significantly increased from the BOTs group to the early-stage OC group. The advanced-stage OC group had significantly lower PNI values compared to the early-stage OC group but significantly higher levels of CA125, HE4, SII, NLR, and FAR. Moreover, the OC group with lymph node metastasis exhibited significantly higher levels of CA125, HE4, SII, NLR, PLR, and FAR, in contrast to the non-metastatic group, while PNI levels were significantly lower. Categorical factors, such as histological grade and pathological classification, showed noticeable discrepancies in CA125 and HE4 levels. NLR was significantly different among the pathological type groups. Among the six inflammatory-nutritional markers, the FAR displayed the greatest diagnostic value. In the analysis of logistic regression, it was observed that a combination marker containing all six inflammatory-nutritional markers exhibited a notably higher AUC value (0.881; 95% CI, 0.823 - 0.926) than any of the individual marker.

**Conclusion:**

PNI, NLR, PLR, MLR, SII, and FAR showed excellent diagnostic performance for OC. The combination of these markers demonstrated a superior diagnostic capability compared to each individual one. The systemic inflammatory indicators may be helpful to diagnose OC.

## 1 Introduction

Ovarian cancer (OC) is often cited as the leading cause of death among gynecologic cancers, with a five-year survival rate of 47% after diagnosis (1). Worldwide, each year approximately 230,000 women are diagnosed with epithelial ovarian cancer (EOC), and 150,000 die due to complications from the disease (2). Despite ongoing advancements in clinical diagnosis and treatment, the outlook for patients with OC remains unsatisfactory. Patients diagnosed with advanced OC have a much lower five-year survival rate (below 30%) than those in the early stages (95%) (3). Therefore, the early detection of OC is important for improving patient prognosis. However, the lack of appropriate screening methods and the asymptomatic growth of the cancer result in OC often being diagnosed only when it reaches an advanced stage. As such, there is a pressing need for dependable indicators to aid in the timely detection and treatment decisions for both patients and physicians. Until now, no single test has provided a reliable indicator of OC. The best biological diagnostic tool for OC will likely be a combination of biomarkers.

One of the most well-known uses for CA125 is as a tumor marker, commonly utilised for monitoring EOC and distinguishing malignant from benign pelvic masses (4, 5). Nonetheless, in the early stages of OC, this marker exhibits a low sensitivity (6). Furthermore, it is prone to producing a high number of false-positive results in non-cancerous gynecological issues like acute pelvic inflammation, adenomyosis, uterine myoma, and endometriosis (7). The possibility of false-positive results must be taken seriously, as it can cause a significant and unnecessary mental and treatment strain for women who are not diagnosed with OC.

In order to improve the precision of detecting OC, new biomarkers like HE4 have been introduced. The levels of HE4, a non-specific tumor marker, can vary in cervical, endometrial, ovarian, and nonepithelial tumors (8). It has been reported that OC tissues exhibit an increased expression of this biomarker (9). Although CA125 and HE4 are the leading markers, they are not powerful enough for early detection of OC (10–12). Extensive research is being conducted to identify new biomarkers that can improve the accuracy and speed of detecting OC for better treatment outcomes. In recent years, mounting evidence has shown that systemic inflammation is a major contributor to the development and advancement of cancer, resulting in a higher chance of metastasis and a weaker response to adjuvant chemotherapy (13). The presence of chronic inflammation is a major contributing factor in the growth and spread of multiple types of solid tumors, such as OC. The relationship between nutrition and inflammation in cancer patients has received considerable attention in the last several years. According to research, the inflammatory microenvironment of cancer is heavily influenced by certain components found in peripheral blood, such as neutrophils, lymphocytes, monocytes, platelets, albumin, globulin, and fibrinogen (14).

Preoperative inflammatory markers, such as systemic immune-inflammation index (SII), prognostic nutritional index (PNI), fibrinogen-to-albumin ratio (FAR), neutrophil-to-lymphocyte ratio (NLR), platelet-to-lymphocyte ratio (PLR) and monocyte-to-lymphocyte ratio (MLR) have been the subject of numerous studies on OC and Multiple studies have deemed them significant prognostic indicators in OC, although only a handful have specifically examined their role in predicting malignancy before surgery (15–18). As far as we know, there is a lack of published research that investigate the efficacy of CA125, HE4, SII, PNI, FAR, NLR, PLR, and MLR in predicting ovarian cancer before surgery. The main focus of this research was to assess the diagnostic potential of CA125, HE4, SII, PNI, FAR, NLR, PLR, MLR, and a combination of six inflammatory-nutritional markers, with the goal of creating a reliable diagnostic index for early detection of OC. In-depth research was performed to ascertain the connection between these markers and the pathological aspects of OC, offering valuable insights for the timely diagnosis and control of this disorder.

## 2 Materials and methods

### 2.1 Criteria for inclusion and exclusion

This is a retrospectively designed study performed in the Hebei General Hospital in HeBei, China. The study included the medical records of patients who had surgery in the Department of Gynecology from 1 January 2019 to 1 December 2022. The study was approved by the ethics committee of the Hebei General Hospital and followed the guidelines set forth in the Declaration of Helsinki.

In this study, 170 patients with OC or benign ovarian tumors (BOTs) were included. They were classified into two groups, with the assistance of two senior pathologists, based on the postoperative pathological results. The two groups consisted of 87 individuals with OC and 83 with BOTs. No chemotherapy or radiation therapy was given to patients with OC before their surgery. The OC clinical stage was established using the International Federation of Gynaecology and Obstetrics (FIGO) criteria. All enrolled individuals with OC underwent a thorough staging process, which consisted of a total hysterectomy, adnexectomy, complete pelvic/para-aortic lymphadenectomy, and peritoneal cytology. The study excluded individuals with infectious illnesses, autoimmune disorders, severe liver or kidney issues, thrombotic conditions, benign or malignant tumors, those who were pregnant, and individuals with preexisting blood disorders.

### 2.2 Clinical and laboratory data collection

The data analysis included several clinical and laboratory parameters such as age, pathological type, FIGO staging, tissue differentiation degree, presence or absence of lymph node metastases, albumin, fibrinogen, neutrophil count, monocyte count, platelet count, lymphocyte count, CA125, and HE4. Prior to the operation, several tests were conducted and recorded within a week, including albumin, fibrinogen, neutrophil count, monocyte count, platelet count, lymphocyte count, and serum tumor biomarkers. The preoperative levels of CA125 and HE4 were measured using a COBAS E602 analyzer (Roche, Switzerland) and the supplied chemiluminescent reagent kit from Roche. Two expert pathologists conducted a thorough review of the pathological examinations. The ETHICS Committee of Hebei General Hospital has given approval for the study.

### 2.3 Inflammation-related markers

The equation used to calculate the serum inflammation-related markers was: FAR= fibrinogen(g/L)/albumin(g/L); PNI = albumin (g/L) + 5 × lymphocyte counts (10^9^/L); SII= platelet count (10^9^/L) × neutrophil count (10^9^/L)/lymphocyte count (10^9^/L); NLR= neutrophil count (10^9^/L)/lymphocyte count (10^9^/L); PLR= platelet count (10^9^/L)/lymphocyte count (10^9^/L); MLR= monocyte count (10^9^/L)/lymphocyte count (10^9^/L).

### 2.4 Statistical analysis

Descriptive statistical analysis was carried out with the help of IBM SPSS statistics V26.0, GraphPad Prism 9.0 software, and R Environment for Statistical Computing software (R Foundation for Statistical Computing). Statistical significance was set at the 5% level and 2-sided p-values were calculated. Normal distribution of variables was assessed using the Shapiro-Wilk test and histograms. The variables that follow a normal distribution were expressed as mean ± standard deviation. For continuous variables, parameters that followed a normal distribution were analyzed by analysis of variance and reported as mean ± standard deviation (SD), and the median and interquartile range will be displayed for those who do not have a normal distribution. The Kruskal-Wallis test was utilized to compare variables across the groups, followed by the Mann-Whitney U test for conducting multiple comparisons. The area under the receiver operating characteristic (ROC), 95% confidence interval (CI), sensitivity, specificity, positive predictive value (PPV), negative predictive value (NPV), positive likelihood ratio (LR), negative LR, and accuracy for the defined variables were calculated to test the diagnostic performance for OC prediction by ROC analysis. Based on Youden’s index from the ROC curve, the optimal cut-off value of the parameters was determined. The connections between important factors were examined through Spearman’s rank correlation and logistic regression analysis. Afterward, a decision curve analysis (DCA) was performed to identify the most effective single parameter for distinguishing between BOTs and OC. In conducting this visual analysis, we utilized specialized DCA software. All calculations were executed according to the method outlined by Vickers and Elkin (19).

## 3 Results

### 3.1 The BOTs group and OC group exhibited significant differences in the levels of CA125, HE4, SII, PNI, FAR, NLR, PLR, and MLR

The final analysis included a total of 87 patients with OC and 83 with BOTs. **Table 1** contains comprehensive data on the laboratory measurements of the research participants.

**Table 1:** Comparison of defined variables between ovarian cancer and benign ovarian tumors.

Variables	Ovarian cancer, median (IQR)	Benign tumor, median (IQR)	Reference level	Z-value	P-value
Number	87	83			
N (10^9^/L)	4.20 (1.97)	3.45 (2.02)	1.8-6.3	-3.361	0.001
L (10^9^/L)	1.41 (0.68)	1.72 (0.68)	1.1-3.2	-3.378	0.001
M (10^9^/L)	0.28(0.16)	0.25(0.10)	0.1-0.6	-2.594	0.009
PLT (10^9^/L)	311.00 (125.00)	258.00 (92.00)	125-350	-3.248	0.001
Alb(g/L)	39.70 (5.97)	42.60 (4.60)	40-55	-3.451	0.001
Fib(g/L)	3.59 (1.54)	2.73 (0.85)	2-4	-6.50	< 0.001
SII	884.86 (859.31)	567.82 (354.31)	/	-5.379	< 0.001
PNI	46.40 (8.10)	51.20 (5.10)	/	-4.322	< 0.001
FAR	0.087 (0.049)	0.062 (0.017)	/	-6.586	< 0.001
NLR	3.10(2.14)	2.15(1.09)	/	-5.123	< 0.001
PLR	211.89(149.75)	153.92(65.52)	/	-4.983	< 0.001
MLR	0.21(0.15)	0.15(0.06)	/	-5.014	< 0.001
CA125 (U/ml)	228.50 (777.87)	16.43 (13.85)	0–35	-8.780	< 0.001
HE4 (pmol/L)	180.20 (308.80)	45.90 (17.22)	premenopause < 70 postmenopause< 140	-8.080	< 0.001

N, absolute neutrophil count; L, absolute lymphocyte count; M, monocyte count; PLT, blood platelet count; Alb, albumin; Fib, fibrinogen; FAR, fibrinogen(g/L)/ albumin(g/L); PNI, albumin (g/L) + 5 × lymphocyte count (10^9^/L); SII, platelet count (10^9^/L) × neutrophil count (10^9^/L)/ lymphocyte count (10^9^/L); NLR, neutrophil count (10^9^/L)/ lymphocyte count (10^9^/L); PLR, platelet count (10^9^/L)/ lymphocyte count (10^9^/L) ,MLR, monocyte count (10^9^/L)/ lymphocyte count (10^9^/L); IQR, interquartile range.

**Figure 1:**
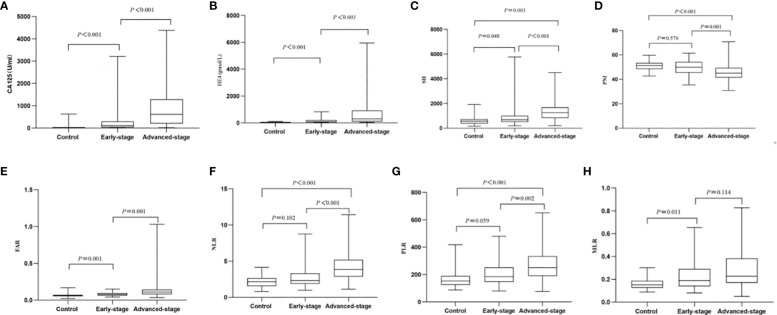
Box of the levels of the eight markers in BOTs group (controls) as well as early-stage OC (Stage I-II) group and advanced-stage OC (Stage III-IV) group. **(A)** CA125; **(B)** HE4; **(C)** SII; **(D)** PNI; **(E)** FAR; **(F)** NLR; **(G)** PLR; **(H)** MLR.

The age range of patients with OC was 21‐76 years, and of patients with BOTs was 26‐78 years. The two groups did not differ significantly in age. **Table 1** presents evidence of a notable difference in absolute neutrophil count, absolute lymphocyte count, absolute monocyte count, blood platelet count, albumin, fibrinogen, SII, PNI, FAR, NLR, PLR, MLR, CA125, and HE4 between BOTs and OC patients (*P*=0.001, *P*=0.001, *P*=0.009, *P*=0.001, *P*=0.001, *P*<0.001, *P*<0.001, *P*<0.001, *P*<0.001, *P*<0.001, *P*<0.001, *P*<0.001, *P*<0.001, *P*<0.001, respectively). Patients with OC showed significantly higher levels of the SII, FAR, NLR, PLR, MLR, CA125, and HE4 [884.86 (859.31), 0.087 (0.049), 3.10(2.14), 211.89(149.75), 0.21(0.15), 228.50 (777.87), 180.20 (308.80)], compared with the patients with BOTs [884.86 (859.31), 0.087 (0.049), 3.10(2.14), 211.89(149.75), 0.21(0.15), 228.50 (777.87), 180.20 (308.80)]. On the other hand, the PNI values were notably lower in the OC group [46.40 (8.10)] in comparison to the BOTs group [51.20 (5.10)]. All markers showed significant diagnostic utility. The PNI, NLR, and PLR levels in the BOTs group did not differ significantly from those of the early-stage OC (Stage I-II) group (*P*=0.578, *P*=0.102, *P*=0.059). Nevertheless, there was a significant increase in CA125, HE4, SII, FAR, and MLR levels observed from the BOTs group to the early-stage OC group (*P*<0.01, *P*<0.01, *P*=0.048, *P*=0.001) (**Figure 1**).

### 3.2 Correlation between CA125, HE4, SII, PNI, FAR, NLR, PLR, MLR, and clinic-pathological characteristics of OC patients

**Table 2** contains a comprehensive analysis of the histopathology and characteristics of the OC-enrolled patients, incorporating differentiation grades and cancer stages. The advanced-stage OC (Stage III-IV) group showed significantly higher levels of CA125 [107.09(267.10)vs. 618.40(1086.20), (early-stage) vs. (advanced-stage), *P*<0.001], HE4 [82.80(153.37)vs. 299.00(816.95), (early-stage) vs. advanced-stage), *P*<0.001], SII [685.64(511.86)vs. 1252.37(857.18), (early-stage) vs. (advanced-stage), *P*<0.001], NLR [2.33(1.42)vs. 3.85(2.32), (early-stage) vs. (advanced-stage), *P*<0.001], PLR [184.19(107.27)vs. 251.25(144.11), (early-stage) vs. (advanced-stage), *P*= 0.002], and FAR [0.08(0.04)vs. 0.11(0.06), (Stage I-II) vs. (advanced-stage), *P*=0.001] compared to the early-stage OC group, while the levels of PNI [49.90(8.77)vs. 45.05(8.08), (early-stage) vs. (advanced-stage), *P*=0.001]were significantly lower in the advanced OC group. Nevertheless, there was a non-significant inclination for MLR levels to rise as OC progressed from early-stage to advanced-stage (**Figure 1**, **Table 2**).

**Table 2:** Relationship between laboratory variables and clinic-pathological characteristics of OC patients.

Variables	N (%)	CA125(U/ml), median (IQR)	HE4 (pmol/L), median (IQR)	SII, median (IQR)	PNI, median (IQR)	FAR, median (IQR)	NLR, median (IQR)	PLR, median (IQR)	MLR, median (IQR)
Age									
≤50	30(34.48%)	329.90(878.63)	174.30(317.61)	795.75(797.02)	49.13(6.85)	0.08(0.04)	2.86(1.89)	202.25(131.63)	0.20(0.16)
> 50	57(65.52%)	215.80(683.83)	180.20(329.31)	947.76(938.70)	45.85(9.42)	0.09(0.06)	3.11(2.40)	223.73(156.81)	0.22(0.16)
Z-value		-0.040	-0.621	-0.406	-1.072	-0.380	-0.388	-0.594	-0.956
P-value		0.968	0.535	0.685	0.284	0.704	0.698	0.553	0.339
FIGO staging									
I-II	42(48.28%)	107.09(267.10)	82.80(153.37)	685.64(511.86)	49.90(8.77)	0.08(0.04)	2.33(1.42)	184.19(107.27)	0.19(0.15)
III-IV	45(51.72%)	618.40(1086.20)	299.00(816.95)	1252.37(857.18)	45.05(8.08)	0.11(0.06)	3.85(2.32)	251.25(144.11)	0.22(0.21)
Z-value		-4.672	-4.196	-3.610	-3.462	-3.381	-3.657	-3.083	-1.580
P-value		<0.001	<0.001	<0.001	0.001	0.001	<0.001	0.002	0.114
Histological grade									
G1	29(33.33%)	121.15(490.74)	66.99(151.25)	829.65(779.16)	46.23(12.94)	0.09(0.07)	3.01(1.63)	195.55(154.37)	0.19(0.15)
G2-G3	58(66.67%)	307.00(784.60)	206.90(339.60)	947.76(931.14)	46.90(6.52)	0.09(0.05)	3.23(2.27)	216.35(144.45)	0.21(0.14)
Z-value		-1.972	-3.134	-1.054	-0.232	-0.790	-1.163	-0.972	-0.763
P-value		0.049	0.002	0.292	0.817	0.429	0.245	0.331	0.445
Pathological type									
Serous	68(78.15%)	294.40(777.60)	204.65(363.93)	962.18(928.09)	46.08(7.18)	0.09(0.05)	3.24(2.30)	224.45(147.75)	0.22(0.14)
Mucinous	8(9.20%)	32.21(177.89)	52.43(39.66)	704.91(729.60)	54.00(14.78)	0.08(0.06)	2.03(1.72)	161.99(152.88)	0.19(0.19)
Clearcell	5(5.75%)	29.28(342.21)	39.10(82.82)	676.47(652.84)	49.70(16.15)	0.09(0.08)	2.12(1.63)	183.71(166.79)	0.17(0.18)
others	6(6.90%)	675.20(2182.28)	138.35(674.45)	743.06(3499.84)	47.29(18.48)	0.08(0.07)	2.71(8.02)	175.87(159.04)	0.15(0.17)
H(K)		12.300	18.219	4.734	3.530	0.972	7.960	3.008	3.173
P-value		0.006	<0.001	0.192	0.317	0.808	0.047	0.390	0.366
Lymph nodes									
Negative	56(64.37%)	161.90(345.05)	84.65(165.88)	711.42(552.86)	49.45(7.45)	0.08(0.03)	2.73(1.62)	190.16(91.63)	0.20(0.15)
Positive	31(35.63%)	714.50(1074.40)	331.00(859.80)	1332.02(999.87)	44.45(5.20)	0.12(0.08)	4.00(2.43)	284.21(199.83)	0.22(0.22)
Z-value		-4.431	-4.768	-3.873	-3.036	-3.341	-3.696	-3.873	-1.507
P-value		<0.001	<0.001	<0.001	0.002	0.001	<0.001	<0.001	0.132

SII, platelet count (10^9^/L) × neutrophil count (10^9^/L)/ lymphocyte count (10^9^/L); PNI, albumin (g/L) + 5 × lymphocyte count (10^9^/L); FAR, fibrinogen(g/L)/ albumin(g/L); NLR, neutrophil count (10^9^/L)/ lymphocyte count (10^9^/L); PLR, platelet count (10^9^/L)/ lymphocyte count (10^9^/L); MLR, monocyte count (10^9^/L)/ lymphocyte count (10^9^/L); IQR, interquartile range. Others: immature teratoma (one case), granulosa cell tumor (one case), endometrioid carcinoma (two cases), carcinosarcoma (two case).

Additionally, the lymph node metastasis OC group showed considerably higher levels of CA125 [714.50(1074.40)vs. 161.90(345.05), (lymph node metastasis) vs. (non-lymph node metastasis), *P*<0.001], HE4 [331.00(859.80)vs. 84.65(165.88), (lymph node metastasis) vs. (non-lymph node metastasis), *P*<0.001], SII [1332.02(999.87)vs. 711.42(552.86), (lymph node metastasis) vs. (non-lymph node metastasis), *P*<0.001], NLR[4.00(2.43)vs. 2.73(1.62), (lymph node metastasis) vs. (non-lymph node metastasis), *P*<0.001], PLR [284.21(199.83)vs. 190.16(91.63), (lymph node metastasis) vs. (non-lymph node metastasis), *P*<0.001], and FAR [0.12(0.08)vs. 0.08(0.03), (lymph node metastasis) vs. (non-lymph node metastasis), *P*=0.001] compared to the non-lymph node metastasis OC group, while PNI levels [44.45(5.20)vs. 49.45(7.45), (lymph node metastasis) vs. (non-lymph node metastasis), *P*=0.002] were significantly lower. Nevertheless, there were no notable differences in the MLR values between the two groups (**Table 2**). The findings implied that increased CA125, HE4, SII, NLR, PLR, and FAR levels before surgery, as well as decreased PNI levels, are indicative of a greater likelihood of advanced ovarian cancer progression and lymph node metastasis. Following that, we explored variances in the variables based on age, histological grade, and pathological category. No statistically significant disparities in the variables were observed among the different age categories, as shown in **Table 2** (*P*= 0.968, *P*= 0.535, *P*= 0.685, *P*= 0.284, *P*= 0.704, *P*= 0.698, *P*= 0.553, *P*= 0.339, respectively). Nevertheless, there were notable discrepancies between CA125 and HE4 in relation to categorical variables such as histological grades (*P*=0.049, *P*=0.002, respectively) and pathological type (*P*=0.006, *P*<0.001, respectively). NLR was significantly different among the pathological type groups (*P* = 0.047). Further, the levels of SII, PNI, PLR, MLR, and FAR did not significantly differ according to the histological grades (*P*=0.292, *P*=0.817, *P*=0.331, *P*=0.445, *P*=0.429, respectively) or pathological types (*P*=0.192, *P*=0.317, *P*=0.390, *P*=0.366, *P*=0.808, respectively).

### 3.3 Efficiency of CA125, HE4, SII, PNI, FAR, NLR, PLR, and MLR in the diagnosis of OC

**Figure 2:**
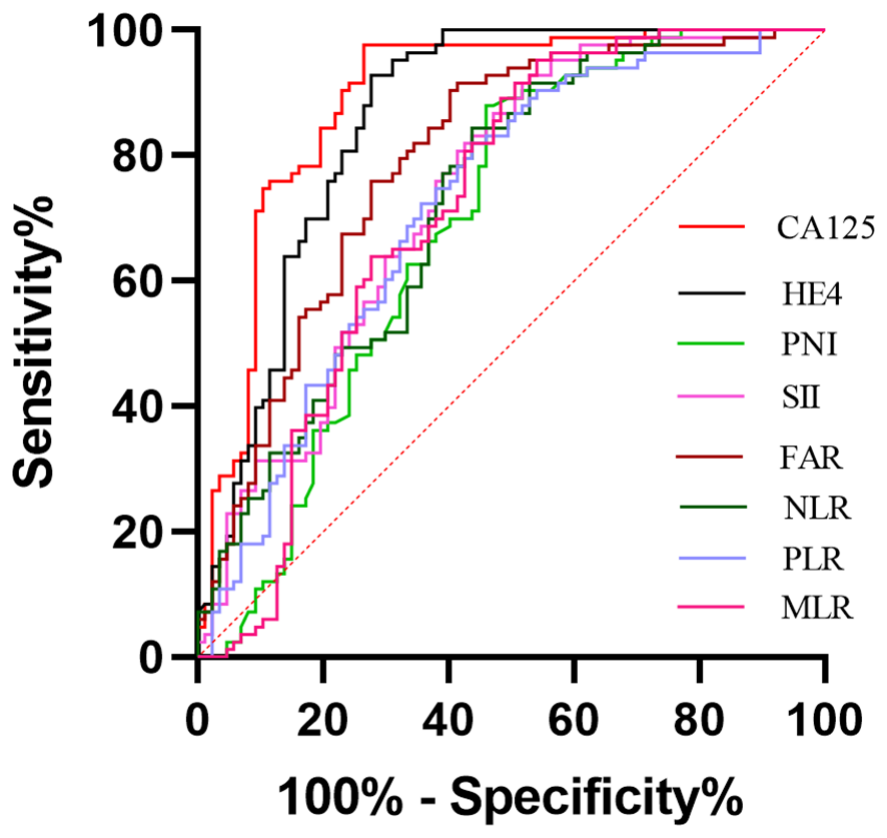
Evaluation of diagnostic performance of the markers for detecting ovarian cancer using receiver operating characteristic curve analysis.

ROC curves were performed to determine the optimal cut-off values, sensitivity and specificity of an independent significant parameter (**Figure 2**). The primary statistics are shown in **Table 3**. The optimum cut-off value was chosen to maximize the Youden index (sensitivity + specificity − 1). The appropriate cut-off value of CA125 (AUC = 0.890, *P*<0.001), HE4 (AUC =0.859, *P*<0.001), FAR (AUC =0.793, *P*<0.001), SII (AUC =0.739, *P*<0.001), NLR (AUC =0.728, *P*<0.001), PLR (AUC =0.721, *P*<0.001), MLR (AUC =0.723, *P*<0.001),and PNI(AUC =0.692, *P*<0.001) for differentiating BOTs and OC were 79.89, 65.16, 0.08, 945.21, 2.92, 201.42, 0.21, and 46.90, respectively; with the corresponding sensitivity of 73.56%, 72.41%, 58.62%, 47.13%, 56.32%, 56.32%, 49.43%, and 54.02%, respectively; specificity of 97.59%, 92.77%, 91.57%, 92.77%,84.34%, 81.93%, 91.57%, and 87.95%, respectively; PPV of 95.52%, 90.00%, 88.14%, 85.42%, 77.78%, 75.38%, 86.00%, and 82.14%, respectively; NPV of 77.67%, 76.00%, 68.47%, 62.30%, 64.49%, 63.81%, 63.33%, and 62.39%, respectively; positive LR of 30.667, 10.056, 6.976, 6.542, 3.34, 2.92, 5.86,and 4.90, respectively; negative LR of 0.270, 0.297, 0.452, 0.570, 0.525, 0.541, 0.552, and 0.528, respectively; accuracy of 84.71, 81.76, 75.29, 68.82, 69.41, 68.24, 70.00, and 70.00 respectively. Among the six inflammatory-nutritional variables (FAR, PNI, NLR, PLR, MLR, and SII), the FAR test showed the greatest sensitivity (58.6%), PPV (88.14%), NPV (68.47%), positive LR (6.976), accuracy (75.29), and lowest negative LR (0.452) in distinguishing between BOTs and OC. Significantly, a logistic regression model incorporating all six inflammatory-nutritional markers demonstrated a higher diagnostic strength than each individual marker (AUC, 0.881; 95% [CI], 0.823 - 0.926).

**Table 3:** Cut-off value and diagnostic value of CA125, HE4, SII, PNI, FAR, NLR, PLR, MLR, and the combination of the six inflammatory-nutritional indices in the diagnosis of OC.

Variables	AUC	Cut-off	95%CI	P-value	Sensitivity (%)	Specificity (%)	PPV (%)	NPV (%)	Positive LR	Negative LR	Accuracy (%)
CA125	0.890	79.89	0.833 -0.933	<0.001	73.56	97.59	95.52	77.67	30.67	0.270	84.71
HE4	0.859	65.16	0.797 -0.908	<0.001	72.41	92.77	90.00	76.00	10.06	0.297	81.76
FAR	0.793	0.08	0.724 -0.851	<0.001	58.62	91.57	88.14	68.47	6.98	0.452	75.29
SII	0.739	945.21	0.666 - 0.803	<0.001	47.13	92.77	85.42	62.30	6.54	0.570	68.82
PNI	0.692	46.90	0.617 - 0.761	<0.001	54.02	87.95	82.14	62.39	4.90	0.528	70.00
NLR	0.728	2.92	0.654 -0.793	<0.001	56.32	84.34	77.78	64.49	3.34	0.525	69.41
PLR	0.721	201.42	0.684 - 0.787	<0.001	56.32	81.93	75.38	63.81	2.92	0.541	68.24
MLR	0.723	0.21	0.649 - 0.789	<0.001	49.43	91.57	86.00	63.33	5.86	0.552	70.00
FAR+SII+PNI+NLR+PLR+MLR	0.881	0.488	0.823 - 0.926	<0.001	78.20	88.00	86.08	79.12	6.52	0.248	82.35

SII, platelet count (10^9^/L) × neutrophil count (10^9^/L)/ lymphocyte count (10^9^/L); PNI, albumin (g/L) + 5 × lymphocyte count (10^9^/L); FAR, fibrinogen(g/L)/ albumin(g/L); NLR, neutrophil count (10^9^/L)/ lymphocyte count (10^9^/L); PLR, platelet count (10^9^/L)/ lymphocyte count (10^9^/L); MLR, monocyte count (10^9^/L)/ lymphocyte count (10^9^/L); AUC, area under the curve; CI, confidence interval; PPV, positive predictive value; NPV, negative predictive value; LR, likelihood ratio.

In general, when evaluated individually, the CA125 examination exhibited the highest level of sensitivity (73.56%), specificity (97.59%), PPV (95.52%), NPV (77.67%), positive LR (30.667), accuracy (84.71%), and lowest negative LR (0.270) in distinguishing BOTs from OC.

**Figure 3:**
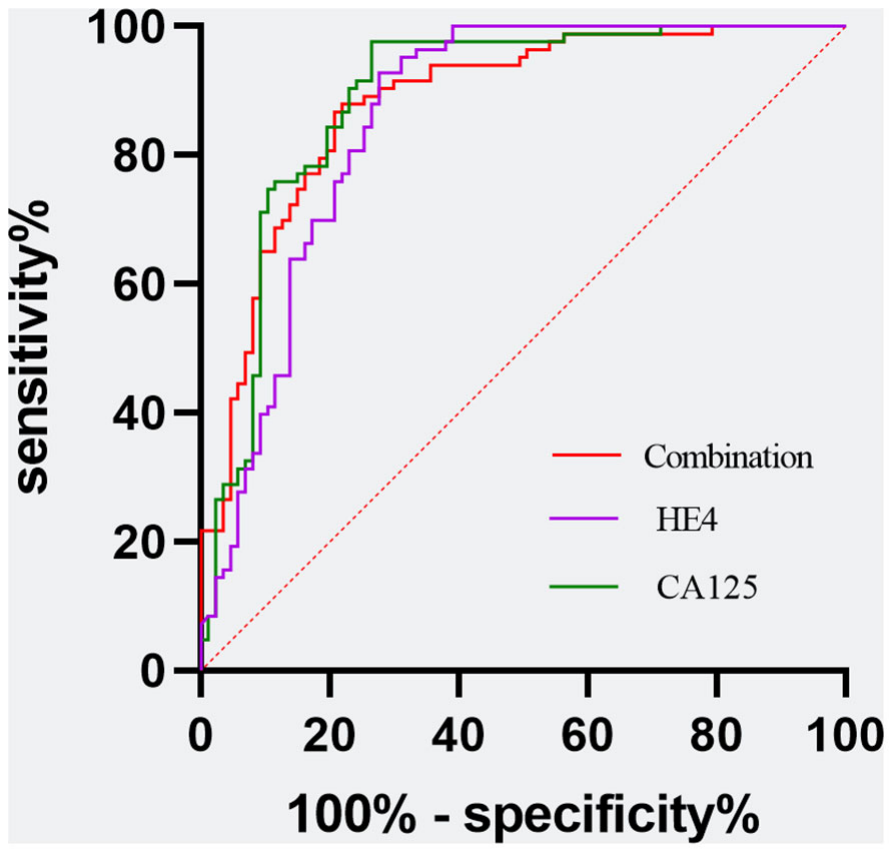
Evaluation of diagnostic performance of the CA125, HE4, and the combination marker for detecting ovarian cancer using receiver operating characteristic curve analysis. Combination marker indicates a logistic regression-based model of SII, PNI, FAR, NLR, PLR, and MLR.

**Figure 4:**
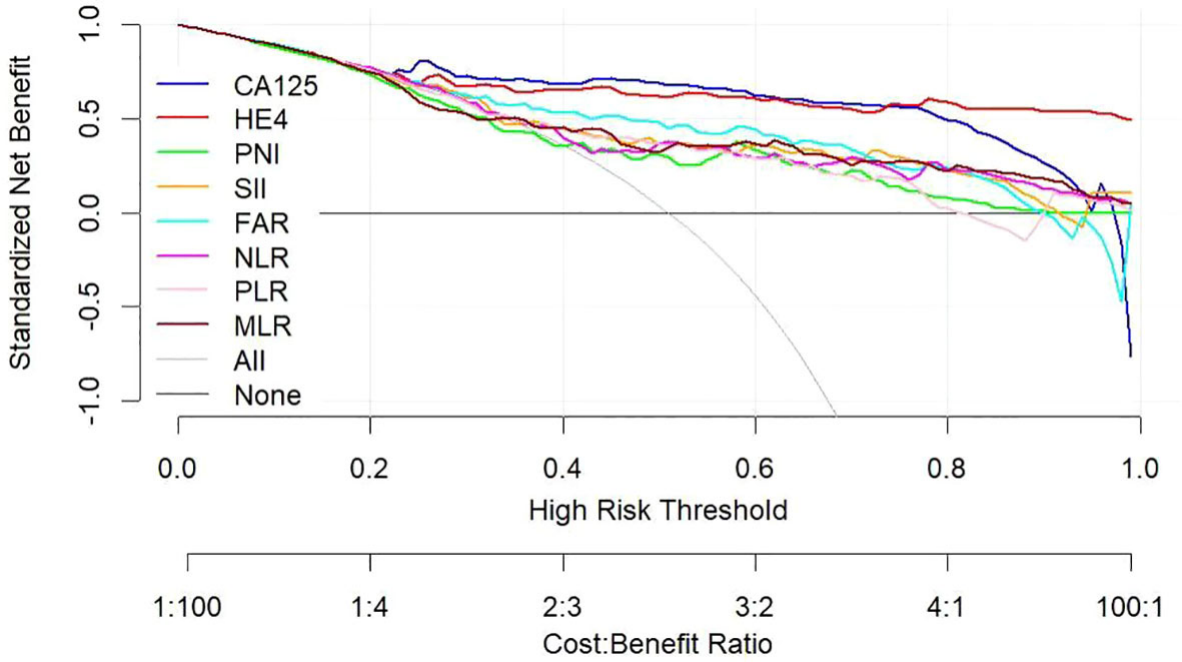
Decision curve showing the net benefit of CA125, HE4, SII, PNI, FAR, NLR, PLR, and MLR in women at risk of developing ovarian cancer. CA125 dosage, positive if ≥79.89 U/mL, negative if <79.89 U/mL; HE4, HE4 dosage, positive if ≥65.16 pmol/L, negative if < 65.16 pmol/L; SII, systemic immune-inflammation index, positive if ≥945.21, negative if <945.21; PNI, prognostic nutritional index, positive if ≤46.9, negative if >46.9; FAR, fibrinogen-to-albumin ratio, positive if ≥0.08, negative if <0.08; NLR, neutrophil-to-lymphocyte ratio, positive if ≥2.92, negative if <2.92; PLR, platelet-to-lymphocyte ratio, positive if ≥201.42, negative if <201.42; MLR, monocyte-to-lymphocyte ratio, positive if ≥0.21, negative if <0.21.

Utilizing a combination of all six inflammatory-nutritional markers in the logistic regression analysis resulted in a significantly higher AUC value (0.881; 95% CI, 0.823 - 0.926) (**Figure 3**). Compared to CA125, the combination marker showed a slight increase in Sensitivity (78.20%), NPV (79.12%), and a decrease in negative LR (0.248) and Accuracy (82.35%), as seen in **Table 3**. The DCA for single CA125, HE4, FAR, PNI, NLR, PLR, MLR, and SII is shown in **Figure 4**. CA125 outperformed all other isolated parameters in terms of clinical utility, as shown in the graphic analysis.

The advanced OC group exhibited significantly higher levels of CA125, HE4, SII, NLR, PLR, and FAR, and significantly lower levels of PNI, when compared to the early-stage OC group. The CA125 test showed the strongest ability to differentiate between BOTs and OC when the defined variables were evaluated individually. By combining all six inflammatory-nutritional indices, the AUC was found to be higher than that of any one index alone. The combination marker showed a slight advantage over CA125, with higher sensitivity and NPV, and lower negative LR and accuracy. The CA125 produced a more favorable result in terms of clinical outcome compared to either HE4 or the combined six inflammatory-nutritional indices. Increased levels of CA125, HE4, SII, NLR, PLR, and FAR prior to surgery may indicate an increased risk of advanced ovarian cancer progression and lymph node metastasis. When compared to other inflammation markers, FAR demonstrated a superior application value.

## 4 Discussion

OC, a highly lethal gynecological cancer, poses a significant global challenge. Due to the absence of distinct early symptoms and reliable screening techniques, OC is typically detected during its advanced stages, leading to relatively high recurrence and mortality rates despite treatment. Timely identification and precise prediction play a crucial role in enhancing survival rates for individuals with OC (20). The use of serum CA125 levels as diagnostic and prognostic biomarkers for OC is common, but solely relying on them may not be sufficient for detecting early OC due to their limited diagnostic ability (21). In various levels, HE4 can be detected as a non-specific tumor marker in cervical, endometrial, ovarian, and nonepithelial tumors (8). According to reports, this biomarker is known to be highly expressed in OC tissues (9). Despite their widespread use, CA125 and HE4 are not reliable enough to detect early-stage OC (10–12). As a result, there has been a significant amount of research focused on discovering new biomarkers for OC.

The initiation and progression of various tumors rely heavily on the immune response and systemic inflammatory processes. They play a crucial role in every aspect of cancer development, from initiating tumors to promoting their spread throughout the body (22). Stephen Paget, a British surgeon, was the first to propose the “seed-soil” theory, which forms the basis of the tumor microenvironment concept. The cellular network is highly intricate, with multiple inflammatory factors produced by both tumor and stromal cells creating an inflammatory microenvironment. The presence of an inflammatory microenvironment significantly impacts the malignant properties of tumors, controlling the biological processes responsible for their growth (23, 24). The inflammatory response is heavily influenced by the body’s immune and nutritional status. Systemic inflammation induced by the progression of cancer is a major contributor to malnutrition and weakened immunity, both of which can significantly decrease survival (25–27). According to studies, malnutrition has been linked to a higher risk of postoperative complications, greater susceptibility to infection, and even a potential increase in tumor recurrence due to weakened tumor immunity (25, 28, 29).

Tumors are often triggered and advanced by uncontrollable inflammation, with changes in inflammatory markers in the blood serving as a reflection of this inflammatory state. Critical markers of inflammation include pro-inflammatory blood cells such as white blood cells (WBC), lymphocytes, monocytes, neutrophils, platelets, and, even more relevant, the MLR, NLR, and PLR. The progression of tumors can be influenced by the release of tumor necrosis factors, interleukin-1, and interleukin-6 from neutrophils (30). Lymphocytes play a vital role in targeting tumors through triggering cytotoxic cell death and hindering the growth and movement of malignant cells (23). Monocytes play a crucial role in the process of tumor occurrence, growth, migration, vascularization, invasion, and metastasis (31). In circulation, platelets can prompt the transformation of tumor cells into an epithelial-mesenchymal state, aiding in their spread to the metastatic site (32). The presence of neutropenia, lymphopenia, monocytosis, and thrombocytosis in cancer leads to inflammation, which can speed up the progression of the disease by promoting angiogenesis, invasion, metastasis, and paraneoplastic phenomena (33–35).

The SII takes into consideration the levels of platelets, neutrophils, and lymphocytes in the blood, serving as an indicator of the multiple Inflammatory and immunological pathways at play in the body. It has outstanding stability (36). It has been utilized in the diagnosis and treatment of various malignant tumors. It is linked to the prediction of patients’ outcomes, reflecting the immune and inflammatory conditions of patients with cancerous tumors (37–39). Although SII is considered an important prognostic factor for OC, its potential as a preoperative indicator of malignancy has not been thoroughly explored in existing research. Albumin is an important marker for both acute-phase proteins and chronic inflammation throughout the body (40). It is frequently utilized to evaluate the body’s nutritional status and is considered a possible prognostic factor for various types of cancer (41, 42). A decrease in serum albumin levels could suggest that the individual is suffering from malnutrition, which can have adverse effects on their overall physical condition. This state of malnourishment can weaken the body’s defense systems, such as cellular immunity, humoral immunity, and phagocytic functioning (43). Preoperative serum albumin levels have been recognized as a significant independent predictor of OS for those with EOC, according to a meta-analysis (44). Elevated levels of plasma fibrinogen, an acute-phase protein, are commonly seen during periods of system-wide inflammation (45). Through its effects on the multiplication and movement of cancer cells, as well as the stimulation of blood vessel growth, it is commonly recognized as a crucial factor in regulating inflammation and cancer progression (46). Numerous studies have consistently demonstrated a correlation between high levels of fibrinogen before treatment and a negative prognosis in a variety of cancers (47).. PNI serves as an indicator for overall inflammatory condition in the body. The combination of serum albumin levels and peripheral blood lymphocyte count, referred to as PNI, provides a comprehensive assessment of the host’s nutritional and immunological conditions. The effectiveness of this approach has been confirmed in accurately predicting both short and long-term prognosis (48). Studies are increasingly showing that preoperative PNI can be served as a prognostic factor for OC patients (48, 49). The results of Miao et al.’s research revealed that PNI is a significant predictor for the overall survival (OS) and progression-free survival (PFS) of OC patients (50). However, it is rarely reported as an indicator of the diagnosis of OC. Researchers have found that FAR, an indicator that takes both fibrinogen and albumin into consideration, can be used to predict outcomes for various malignancies (16, 51). A different strategy for improving the predictive ability of inflammation and nutritional status in patients is to combine fibrinogen and albumin in the FAR. Elevated levels of serum fibrinogen and decreased levels of serum albumin are commonly accepted as reliable indicators of heightened systemic inflammation (52, 53). According to Xie H et al.’s investigation, a high FAR could be an indication of more aggressive tumor characteristics and the development of systemic inflammation (54). Additionally, FAR was observed to heighten the sensitivity of inflammation and nutrition levels in EOC patients, proving to be a superior predictor of EOC prognosis than individual markers like fibrinogen or albumin.

Our study examined the diagnostic utilities of CA125, HE4, and six inflammatory-nutritional markers (PNI, NLR, PLR, MLR, SII, and FAR) in patients with OC. All markers demonstrated significant diagnostic value in identifying patients with OC. CA125, HE4, SII, FAR, and MLR levels significantly increased from the BOTs to early-stage OC groups. Nevertheless, there was no significant difference in PNI, NLR, and PLR levels between the BOTs and early-stage OC groups. The advanced-stage OC group had notably elevated levels of CA125, HE4, SII, NLR, and FAR, in contrast to the early-stage OC group. On the other hand, the advanced OC group had significantly lower PNI values. There was a slight, non-significant trend of MLR levels increasing from the early stages to the advanced stages. Additionally, the presence of lymph node metastasis in the OC group was associated with significantly elevated levels of CA125, HE4, SII, NLR, PLR, and FAR when compared to the OC group without lymph node involvement. Conversely, the PNI values were notably decreased in the OC group with lymph node metastasis. There were no notable differences in the MLR values among the two groups. The findings indicate that elevated preoperative levels of CA125, HE4, SII, NLR, PLR, and FAR, as well as decreased PNI levels, are associated with a higher probability of advanced ovarian cancer progression and lymph node metastasis. Significant variations were observed in CA125 and HE4 levels among categorical variables such as histological grades and pathological type. NLR was significantly different among the pathological type groups. However, the SII, PNI, PLR, MLR, and FAR levels did not significantly differ according to the histological grades or pathological types. Based on the six inflammatory-nutritional indices (FAR, PNI, NLR, PLR, MLR, and SII), the FAR test was identified as the top performer in distinguishing between BOTs and OC, exhibiting the highest sensitivity (58.6%), PPV (88.14%), NPV (68.47%), positive LR (6.976), accuracy (75.29), and lowest negative LR (0.452).

Additionally, the ROC curve analysis revealed significant AUC values for all eight markers, ranked in the following order: CA125 (0.890; 95% CI,0.833 -0.933), HE4 (0.859; 95% CI, 0.797 -0.908), FAR (0.793, 95% CI 0.724 -0.851), SII (0.739, 95% CI 0.666 - 0.803), NLR (0.728, 95% CI 0.654 -0.793), MLR (0.723, 95% CI 0.649 - 0.789), PLR (0.721, 95% CI 0.684 - 0.787), and PNI (0.692, 95% CI, 0.617 - 0.761). The logistic regression analysis revealed that a composite marker comprising all six inflammatory-nutritional markers exhibited a significantly greater AUC value (0.881; 95% CI, 0.823 - 0.926) compared to each individual marker. The combination marker model displayed the most sensitivity (78.20%) compared with CA125 (73.56%), HE4 (72.41%), FAR (58.62%), SII (47.13%), PNI (54.02%), NLR (56.32%), PLR (56.32%), and MLR (49.43%).

Our research revealed that incorporating inflammatory biomarkers alongside CA125 and HE4 is an effective method for differentiating between BOTs and OC. Before surgery, biomarkers can be helpful, but imaging tests are usually the main method of managing abnormalities found in serum biomarkers. By further exploring the inflammatory pathways linked to tumors, we may uncover improved combinations of tumor and inflammatory biomarkers.

This study has a number of shortcomings that warrant mentioning. First, this was a retrospective study conducted at a single medical center, and further research on a larger scale is needed to validate our findings. Secondly, only patients with BOTs were included in the control group. In future studies, it is recommended to include both individuals without health issues and those with the disease as controls. Despite this, our research provides robust evidence base for the reliability and reproducibility of the diagnostic traits under study, as a result of our meticulous inclusion and exclusion standards and highly significant statistical results.

In conclusion, systemic inflammatory indicators (PNI, NLR, PLR, MLR, SII, and FAR) showed excellent diagnostic performance for OC. Significantly, the combination of these markers demonstrated a superior diagnostic capability compared to each individual one. Furthermore, FAR exhibited a greater application value than other inflammation-related markers, including PNI, NLR, PLR, MLR, and SII. Our findings suggest systemic inflammatory indicators may be helpful to diagnose OC. In the near future, we aim to carry out an external validation study.

## Data availability statement

The datasets used and/or analyzed during the current study are available from the corresponding author upon reasonable request.

## Ethics statement

The studies involving humans were approved by the ethics committee of the Hebei General Hospital. The studies were conducted in accordance with the local legislation and institutional requirements. The human samples used in this study were acquired from a by- product of routine care or industry. Written informed consent for participation was not required from the participants or the participants’ legal guardians/next of kin in accordance with the national legislation and institutional requirements.

## Author contributions

LS: Investigation, Software, Supervision, Writing – original draft, Writing – review & editing, Conceptualization, Data curation, Formal analysis, Validation, Resources. QW: Writing – original draft, Writing – review & editing, Conceptualization, Investigation, Methodology, Resources. SB: Conceptualization, Writing – original draft, Formal analysis, Methodology, Project administration. JZ: Formal analysis, Methodology, Resources, Writing – original draft. JQ: Data curation, Investigation, Resources, Writing – original draft. JMZ: Data curation, Project administration, Resources, Writing – original draft.
